# Evaluating the Reliability and Quality of Sarcoidosis-Related Information Provided by AI Chatbots

**DOI:** 10.3390/healthcare13111344

**Published:** 2025-06-05

**Authors:** Nur Aleyna Yetkin, Burcu Baran, Bilal Rabahoğlu, Nuri Tutar, İnci Gülmez

**Affiliations:** Department of Pulmonology, Faculty of Medicine, Erciyes University, 38039 Kayseri, Türkiye; burcubaran@hotmail.com (B.B.); brabah91@hotmail.com (B.R.); drnuritutar@gmail.com (N.T.); incigul@erciyes.edu.tr (İ.G.)

**Keywords:** sarcoidosis, AI chatbots, health information quality, readability, patient education

## Abstract

**Background and Objectives:** Artificial intelligence (AI) chatbots are increasingly employed for the dissemination of health information; however, apprehensions regarding their accuracy and reliability remain. The intricacy of sarcoidosis may lead to misinformation and omissions that affect patient comprehension. This study assessed the usability of AI-generated information on sarcoidosis by evaluating the quality, reliability, readability, understandability, and actionability of chatbot responses to patient-centered queries. **Methods**: This cross-sectional evaluation included 11 AI chatbots comprising both general-purpose and retrieval-augmented tools. Four sarcoidosis-related queries derived from Google Trends were submitted to each chatbot under standardized conditions. Responses were independently evaluated by four blinded pulmonology experts using DISCERN, the Patient Education Materials Assessment Tool—Printable (PEMAT-P), and Flesch–Kincaid readability metrics. A Web Resource Rating (WRR) score was also calculated. Inter-rater reliability was assessed using intraclass correlation coefficients (ICCs). **Results**: Retrieval-augmented models such as ChatGPT-4o Deep Research, Perplexity Research, and Grok3 Deep Search outperformed general-purpose chatbots across the DISCERN, PEMAT-P, and WRR metrics. However, these high-performing models also produced text at significantly higher reading levels (Flesch–Kincaid Grade Level > 16), reducing accessibility. Actionability scores were consistently lower than understandability scores across all models. The ICCs exceeded 0.80 for all evaluation domains, indicating excellent inter-rater reliability. **Conclusions**: Although some AI chatbots can generate accurate and well-structured responses to sarcoidosis-related questions, their limited readability and low actionability present barriers for effective patient education. Optimization strategies, such as prompt refinement, health literacy adaptation, and domain-specific model development, are required to improve the utility of AI chatbots in complex disease communication.

## 1. Introduction

The Internet has become an indispensable source of health-related information for patients worldwide, profoundly influencing their understanding of diseases and treatment decisions [1]. Although online access to medical content offers convenience and broad reach, the quality, reliability, and readability of this information remain highly variable [2]. This issue is especially concerning for conditions such as sarcoidosis, a heterogeneous and often misunderstood multisystem granulomatous disease, in which inaccurate information may contribute to diagnostic delays, increased anxiety, and inappropriate health-seeking behaviors.

In recent years, generative large language models (LLMs), such as ChatGPT and Gemini, have emerged as accessible tools capable of generating human-like responses to a wide array of health-related queries. Their conversational nature and real-time availability have positioned them as promising adjuncts for patient education, especially in contexts where professional consultation is delayed or unavailable. However, despite their growing popularity, concerns persist regarding clinical accuracy, health literacy adaptation, and practical actionability of their outputs.

Sarcoidosis poses a unique challenge to both clinicians and patients. Its protean manifestations across multiple organs often require detailed and nuanced explanations, particularly regarding pulmonary involvement, diagnostic ambiguity, and long-term immunosuppressive treatment. Previous studies have documented the poor quality and limited reliability of sarcoidosis-related content available on public platforms such as YouTube [3]. However, the performance of artificial intelligence (AI) chatbots in delivering trustworthy and patient-relevant information about sarcoidosis remains underexplored [4].

To address this gap, this study evaluated the quality, readability, and actionability of AI chatbot-generated responses to commonly searched sarcoidosis-related queries. By employing standardized metrics and expert assessments, we aimed to determine whether these tools could meaningfully support patient understanding in complex disease contexts.

### Related Work

A growing body of research has evaluated the performance of AI chatbots in disseminating medical information across various domains. In evaluations conducted on appendicitis and Achilles tendon rupture, some chatbots demonstrated high medical accuracy; however, their responses were generally found to have low readability, linguistic density, and variable citation transparency [5,6]. In a comparative analysis, five chatbots were evaluated for erectile dysfunction-related queries using the DISCERN instrument and readability metrics, in order to assess the quality and accessibility of the information provided to patients [7]. The study highlighted inconsistencies across models and emphasized the lack of actionable guidance. Other studies have explored chatbot performance in specific domains, such as vasectomy [8], colorectal cancer [9], and ophthalmic conditions [10], with similar conclusions. Although AI chatbots often deliver factually accurate information, their readability, structure, and alignment with patient-centered communication remain limited.

Sarcoidosis is a rare immune-mediated granulomatous disease characterized by the formation of non-caseating granulomas in the affected tissues. It most commonly involves the lungs and intrathoracic lymph nodes but can affect virtually any organ system, including the skin, eyes, heart, and nervous system. The clinical course of sarcoidosis is highly variable, ranging from asymptomatic and self-limiting forms to chronic progressive disease, with significant morbidity. By focusing on sarcoidosis, a rare and clinically complex disease, our study aimed to evaluate the quality and readability of chatbot-generated responses to frequently searched sarcoidosis-related queries and explore their potential implications for patient education in rare diseases. To this end, we employed four distinct and highly popular questions identified via Google Trends to represent varying levels of informational complexity. We utilized multidimensional evaluation tools, including DISCERN, the Patient Education Materials Assessment Tool—Printable (PEMAT-P), the Web Resource Rating (WRR), and established readability indices.

## 2. Materials and Methods

### 2.1. Study Design

This cross-sectional evaluation study was conducted on 20 May 2025, with the primary objective of assessing the quality, readability, and actionability of the responses generated by a diverse range of AI chatbots to common patient-oriented queries regarding sarcoidosis. To this end, a comparative and descriptive analytical framework was employed. The chatbots included in the analysis represent two major categories: general-purpose conversational platforms and search-augmented information-retrieval tools. Specifically, the evaluated chatbots were: ChatGPT-4 (OpenAI, https://chat.openai.com, accessed on 1 May 2025), ChatGPT-4o and ChatGPT-4o Deep Research (OpenAI, https://openai.com/chatgpt, May 2025 release), Copilot (Microsoft Bing, https://copilot.microsoft.com), DeepSeek and DeepSeek Deep Think (https://www.deepseek.com, accessed on 1 May 2025), Gemini2 Flash (Google, https://gemini.google.com), Grok3 and Grok3 Deep Search (X.AI, https://x.ai/grok), and Perplexity and Perplexity Research (https://www.perplexity.ai, accessed on 1 May 2025).

To ensure rigorous objectivity and reliability, the responses obtained from each chatbot were independently scored by four experienced pulmonology specialists, who were blinded to each other’s evaluations. These evaluators were carefully selected based on their expertise in sarcoidosis management and clinical practice to ensure credible and consistent judgment.

### 2.2. Query Selection and Prompting Procedure

Query selection and prompting procedures were conducted systematically to ensure that the chosen queries accurately reflected authentic patient concerns and maintained strong clinical relevance. To identify the most frequently encountered and clinically meaningful patient inquiries related to sarcoidosis, Google Trends data spanning a 12-month period, from 20 May 2024 to 20 May 2025, were meticulously analyzed. Four distinct queries were selected for a detailed analysis. These queries represent a range of informational complexities, extending from basic disease information to specific clinical management issues. The final set of queries selected for this study included the following: Q1: ‘What are the symptoms of sarcoidosis?’, Q2: ‘How serious is sarcoidosis, and what type of disease is it?’, Q3: ‘What happens when sarcoidosis affects the lungs?’, and Q4: ‘Can sarcoidosis be treated, and will I need medications for life?’. These queries were selected to represent a gradient of informational complexity ranging from basic symptom recognition to long-term management considerations. All queries were utilized exactly as originally identified, preserving their original English wording, thus ensuring standardization and consistency in the assessment of the chatbot-generated responses.

### 2.3. Query Submission Procedure

The queries were individually submitted to each chatbot on the same day (20 May 2025) by a single experienced investigator to maintain consistency and reduce user-induced variability. All queries were manually entered within a controlled timeframe to ensure uniform data-collection conditions.

**Figure 1:**
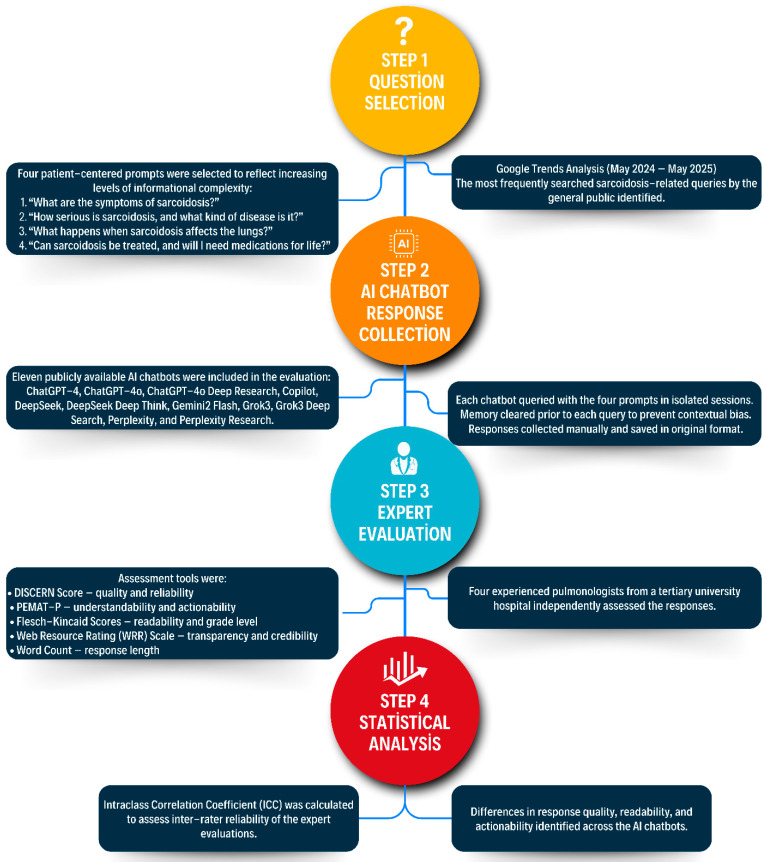
Workflow diagram of the study outlining the four sequential steps: (1) query selection based on Google Trends analysis of commonly searched sarcoidosis-related questions; (2) AI chatbot response collection using standardized prompts and controlled conditions; (3) independent expert evaluation using validated tools (DISCERN, PEMAT-P, Flesch–Kincaid scores, Web Resource Rating, and word count); and (4) statistical analysis of inter-rater reliability and performance differences across chatbot outputs. This process was designed to assess the quality, readability, and actionability of AI-generated information on sarcoidosis.

To mitigate the potential bias arising from search history or personalized web content, the investigator used the Incognito browser mode. The browser cache and cookies were systematically cleared prior to each chatbot session. This precautionary measure ensured that the generated responses reflected the unbiased innate performance of each chatbot. The responses were recorded exactly as generated by the chatbots, without any follow-up queries, clarifications, or modifications, thus accurately reflecting the raw, unaltered output. The overall design and evaluation process of the study are summarized in the flowchart presented in Figure 1.

### 2.4. Data Analysis

Responses obtained from AI chatbots were systematically analyzed using validated instruments specifically selected for their relevance and appropriateness in evaluating health-related information. These instruments have been widely used in previous studies to evaluate both printed and digital patient education materials. The DISCERN instrument was used to assess the content reliability and the overall quality of the responses. PEMAT-P was used to evaluate the understandability and actionability of chatbot-generated content. Readability was assessed using the Flesch–Kincaid Reading Score, which evaluates the ease of comprehension of written material, and the Flesch–Kincaid Grade-1 Level Score, which indicates the approximate education level required to understand the provided responses (Table 1).

We also incorporated the WRR score to provide a composite assessment of the overall quality of the chatbot-generated content. The WRR framework was specifically developed to capture multiple evaluative dimensions that are not fully covered by existing tools. These dimensions include content reliability, structural organization, relevance to queries, citation transparency, and educational value. Each response was scored independently by four blind pulmonology experts using a standardized rubric, and the final WRR score represented the average across rates. This composite measure offers an integrative view of both the formal quality and practical usability of AI-generated medical content, particularly in the context of patient education.

The inter-rater reliability of the four pulmonology specialists was statistically quantified by calculating the intraclass correlation coefficient (ICC), which confirmed the consistency and reproducibility of the evaluations. The ICC was calculated for each metric to evaluate the rating consistency across chatbot responses. The ICC was calculated for each metric to evaluate the rating consistency across chatbot responses. According to established guidelines, ICC values < 0.50 indicate poor reliability, 0.50–0.75 indicate moderate reliability, 0.75–0.90 indicate good reliability, and values > 0.90 indicate excellent reliability. Statistical analyses were conducted using R-4.4.3 to compare chatbot performance and assess expert rating reliability. Quantitative analyses were conducted using descriptive statistics, and inferential tests were performed to explore the significant differences in chatbot performance. All statistical analyses were conducted using the R statistical software (version 4.5.0), with statistical significance defined as a *p*-value of less than 0.05.

## 3. Results

### 3.1. Inter-Rater Reliability Analysis

The inter-rater reliability for each of the four sarcoidosis-related queries was assessed using ICC across three evaluation domains: DISCERN, PEMAT-P, and WRR. All ICC values exceeded the commonly accepted threshold of 0.80, indicating excellent agreement between the four pulmonology experts. The highest consistency was observed for the first query, with ICCs of 0.91 (DISCERN), 0.89 (PEMAT-P), and 0.93 (WRR). Even the lowest ICC value—0.80 for PEMAT-P in the fourth query—demonstrated. 

For the DISCERN tool, which assesses the reliability and quality of medical information, the ICC values ranged from 0.82 (Q4) to 0.88 (Q2), demonstrating stable consensus in rating informational content. PEMAT-P, which evaluates understandability and actionability, showed ICCs between 0.80 (Q4) and 0.89 (Q1), again reflecting strong evaluator alignment. The highest ICC values were observed in the WRR domain, which integrates multiple aspects of quality and relevance; WRR scores ranged from 0.82 (Q4) to 0.93 (Q1), indicating near-perfect agreement for foundational clinical content.

### 3.2. Individual Chatbot Performance

Substantial differences were observed among the 11 AI chatbots across all the evaluated domains, including reliability, understandability, and actionability. ChatGPT-4o Deep Research consistently achieved the highest overall performance across all the evaluation domains. It obtained a mean DISCERN score of 70.06, a PEMAT-P score of 77.75, and a WRR score of 70.78. These findings reflect a high standard of medical reliability accompanied by content that is both actionable and sufficiently understandable to support patient education. Perplexity Research demonstrated similarly robust performance, with mean scores of 68.75 (DISCERN), 76.38 (PEMAT-P), and 82.03 (WRR), ranking first in the WRR domain. These results underscore the utility of retrieval-augmented LLM for producing comprehensive, structured, and contextually appropriate health information.

Grok3 Deep Search also performed well across all domains, with a mean DISCERN score of 63.56, a PEMAT-P score of 82.25, and a WRR score of 70.63. Notably, its PEMAT-P score was among the highest, indicating that its responses were not only informative but also presented in a format that facilitated patient comprehension and actionability.

In contrast, general-purpose models such as Copilot and ChatGPT-4 showed lower overall performance.Copilot had the lowest DISCERN (35.00), PEMAT-P (32.50), and WRR (6.32) scores, reflecting substantial deficiencies in the clarity, reliability, and usefulness of its responses. ChatGPT-4 demonstrated moderate performance, with an average DISCERN score of 48.75 and PEMAT-P and WRR scores of 44.25 and 8.94, respectively, indicating some improvement over Copilot but falling short of models enhanced with research or search capabilities.

The ChatGPT-4o, DeepSeek, and Gemini2 Flash occupied an intermediate performance tier. ChatGPT-4o displayed modest reliability (mean DISCERN = 57.19), with average PEMAT-P and WRR scores of 51.75 and 6.27, respectively. DeepSeek and Gemini2 Flash showed comparable trends, with balanced but unremarkable performance across domains. Although some of these models generated more readable content, they were often deficient in terms of structural clarity, depth, or action-oriented guidance (Table 2).

**Figure 2:**
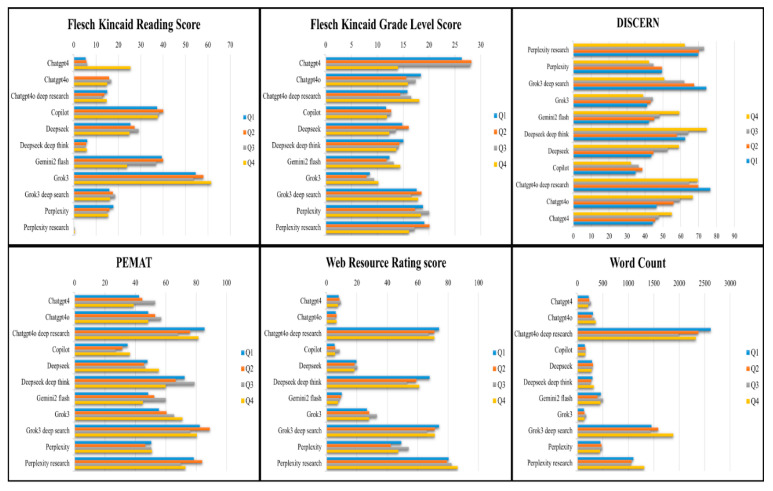
Comparative distribution of chatbot performance metrics across four sarcoidosis-related queries, including readability, quality, actionability, and response length.

Importantly, DeepSeek Deep Think, while not leading in any single metric, maintained high consistency across all domains, with mean scores of 64.50 (DISCERN), 69.25 (PEMAT-P), and 60.70 (WRR), positioning it as one of the most balanced performers. Figure 2 illustrates chatbot performance in terms of readability, reliability, actionability, overall quality, and response length for each query.

### 3.3. Comparative Analysis Based on Chatbot Categories

To examine the effect of chatbot architecture on performance, the models were grouped into general-purpose conversational agents (ChatGPT-4, ChatGPT-4o, Gemini2 Flash, Copilot, and Grok3), and search-augmented or research-enhanced tools (ChatGPT-4o Deep Research, DeepSeek, DeepSeek Deep Think, Grok3 Deep Search, Perplexity, and Perplexity Research).

The search-augmented models outperformed their general-purpose counterparts across all key domains. They achieved higher mean scores for the DISCERN (66.3 vs. 45.9), PEMAT-P (69.4 vs. 48.1), and WRR (66.3 vs. 8.8), indicating superior reliability, actionability, and structural clarity.

However, general-purpose chatbots demonstrate better readability. Their average Flesch–Kincaid Reading Score was higher (35.6 vs. 13.7) and the Grade Level Score was lower (11.9 vs. 16.9), suggesting more accessible language. Word counts also differed substantially; search-augmented models produced longer responses (mean = 1601.6 words) than general-purpose models (mean = 221.8 words) (Table 2).

## 4. Discussion

This study presents a systematic evaluation of 11 AI chatbots in terms of their capacity to provide reliable, understandable, and actionable responses to four frequently searched questions about sarcoidosis. Using validated tools, such as DISCERN, PEMAT-P, Flesch–Kincaid readability metrics, and a composite WRR, the quality and usability of chatbot-generated content were assessed across multiple domains. Their findings revealed substantial variations in the chatbot performance. Models incorporating retrieval-augmented or research-enhanced architectures, such as ChatGPT-4o Deep Research, Perplexity Research, and Grok3 Deep Search, achieved the highest median scores across DISCERN, PEMAT-P, and WRR. These chatbots produced detailed, structured, and evidence-consistent responses, indicating their potential value in patient education for complex conditions such as sarcoidosis. Conversely, the general-purpose conversational agents, ChatGPT-4, ChatGPT-4o, Copilot, and Grok3, exhibited lower reliability and limited actionability. Although these models often generate more readable content, as measured by higher Flesch Reading Ease scores and lower Grade Level estimates, their outputs were typically less comprehensive and lacked clear guidance for patients. Copilot consistently underperformed across all the dimensions (Table 3).

An important strength of this study was the excellent inter-rater reliability observed across all evaluated domains. The high ICC values (>0.80) obtained for the DISCERN, PEMAT-P, and WRR scoring demonstrated consistent agreement among the four blinded pulmonologists, despite the subjective nature of the qualitative assessments. This high level of inter-rater agreement supports the reliability of the evaluation framework and provides further assurance regarding the consistency of the findings. Particularly noteworthy is the high agreement, even for more nuanced and complex chatbot responses, indicating that subtle quality differences were reliably detected by the domain experts.

A key finding of this study is the inverse relationship between content quality and readability. Retrieval-augmented models delivered responses with superior structure and medical reliability, but at advanced reading levels (mean FKGL > 16), potentially limiting accessibility for individuals with lower health literacy. Conversely, more readable outputs from general-purpose models often lack depth of information or actionable content. This trade-off highlights a critical barrier in chatbot-mediated health communication: achieving both accuracy and accessibility remains challenging, especially for rare and complex diseases, such as sarcoidosis.

These observations are consistent with those of prior studies that assessed the performance and limitations of large language models in healthcare communication. Multiple investigations have demonstrated that AI-generated content, particularly that produced by ChatGPT, tends to score highly in reliability and structural coherence yet often falls short in terms of accessibility due to elevated reading complexity. For instance, ChatGPT-generated responses to appendicitis-related queries were found to be more reliable than those of competing models but were consistently written at a higher reading level, thus reducing their comprehensibility to the public [11]. A similar pattern was reported in the context of Achilles tendon rupture, where GPT-4-based content achieved strong DISCERN scores yet remained linguistically dense and better suited for college-educated readers [6]. These studies reinforce the notion that high-quality informational content generated by LLMs frequently exceeds the average health literacy of the target patient population. Comparable findings have also emerged in specialty domains such as spine surgery. In an evaluation of GPT-4 responses to common patient questions in that field, the model again surpassed traditional online sources in terms of reliability. However, readability scores indicated a substantial barrier to understanding lay users. The mean Flesch–Kincaid Grade Level for the GPT-4 responses approached 13, implying that a high school graduate would likely struggle to fully comprehend the text without additional support [12]. This persistent trade-off between informational depth and linguistic simplicity is a critical challenge in AI-driven patient education. Recent studies have suggested that such limitations can be modified through prompt optimization. When GPT-4 was explicitly instructed to produce layperson-friendly summaries, it demonstrated a marked improvement in readability while maintaining moderate scientific fidelity. This implies that model behavior can be partially directed through carefully constructed prompts, offering a pathway toward more accessible AI-generated health content without compromising accuracy or coherence. While improving readability is essential, the actionability of chatbot responses, that is, their capacity to offer practical, patient-oriented recommendations, remains another under-addressed area. Our observation that even high-performance chatbots often fall short of delivering clear, actionable guidance aligns with the previous systematic reviews of adjacent domains. For example, while many health communication tools are designed to be understandable and well structured, relatively few provide patients with clear, personalized actions that can be readily implemented in real-life decision making [10]. Similarly, an analysis of online resources for dementia caregivers found that most materials lacked user-centered co-design and were written at reading levels exceeding what would be considered accessible, thus diminishing their real-world applicability despite being technically accurate [13]. These findings collectively suggest that understandability alone does not guarantee clinical usefulness; the content must also be actionable and tailored to users’ needs. It should also be noted that tools such as DISCERN and PEMAT-P, when validated for traditional health education materials, may not fully accommodate the evolving structural and interactive nature of AI-generated responses. Therefore, their application to chatbot outputs should be cautiously interpreted. Together, these studies illustrate a broader structural limitation of the current generation of AI chatbots: the difficulty of simultaneously optimizing reliability, readability, and actionability. Most models still require a trade-off: prioritizing one dimension tends to occur at the expense of another. In sarcoidosis, a condition that requires nuanced explanations regarding organ-specific manifestations, diagnostic uncertainty, and long-term management, such imbalances may be particularly consequential. The complexity inherent to sarcoidosis magnifies the importance of delivering content that is not only factually correct and linguistically accessible but also organized in a way that supports informed decision making and shared care planning. These findings underscore the need for further innovation in AI chatbot design. One promising avenue involves tailoring model outputs through advanced prompt engineering, which dynamically adjusts user literacy levels and preferences. Additionally, the development of domain-specific guideline-informed AI models, particularly for rare or complex diseases, may be necessary to ensure both accuracy and contextual relevance. These specialized models could integrate structured medical knowledge bases and clinical pathways to produce responses that are not only trustworthy but also actionable and appropriate for varying user profiles.

The unique clinical characteristics of sarcoidosis pose additional challenges to AI-powered chatbot systems. Owing to its multisystemic nature, educational content must encompass a broad range of organ-specific manifestations, including pulmonary, dermatologic, ocular, cardiac, and neurological involvement. However, the current AI models often struggle to address organ-specific presentations and their respective clinical priorities in a sufficiently integrated and balanced manner. Furthermore, the clinical management of sarcoidosis frequently involves “watchful waiting” strategies, individualized treatment plans, and long-term follow-up decisions. Consequently, AI-generated prognostic guidance tends to rely on generalized patterns rather than evidence-based guidelines, which may lead to either excessive uncertainty or a false sense of certainty in patients.

In diseases, such as sarcoidosis, in which the distinction between active inflammation and fibrotic progression is clinically critical, AI-generated responses that fail to adequately differentiate between these two states may result in misleading guidance. Additionally, because AI models are often trained on datasets that lack a sufficient volume of high-quality, expert-informed, and up-to-date content, they may exhibit significant informational gaps and a reduction in source reliability. Responses pertaining to nonsteroidal treatment options, immunosuppressive agents, biologics, or imaging strategies used in follow-up are often outdated or inconsistent with the current clinical guidelines. Of particular note is the near-complete absence of any reference to antifibrotic therapies in AI-generated outputs, despite their increasing relevance in advanced pulmonary sarcoidosis.

These limitations underscore the need for AI-based health communication tools developed for sarcoidosis to be tailored not only for factual medical accuracy but also for contextual awareness, multidisciplinary scope, and sensitivity to patient-specific needs. Purpose-built, domain-specific AI models integrated with structured clinical knowledge bases and current guidelines may provide a more robust and clinically meaningful framework for supporting patients with rare and complex diseases, such as sarcoidosis.

This study has several limitations. An additional consideration not explicitly addressed is the potential limitation of the available high-quality, structured sarcoidosis content on the open web, which may restrict the representativeness of the chatbot training data. Unlike more prevalent diseases, sarcoidosis is underrepresented in digital health sources, possibly influencing the quality of AI-generated responses. Future studies should explore the origin, quantity, and classification of online content related to sarcoidosis in order to better characterize the informational gaps and limitations of current AI-based models. First, chatbot responses were evaluated only in English, limiting generalizability to non-English-speaking populations. Second, only four fixed queries were used, which may not capture the full variability in user inputs. Third, the assessments were based on expert reviews rather than real patient interactions, potentially overlooking user experience factors. Finally, while the WRR provides a useful composite measure, it lacks external validation and includes subjective components. Moreover, although the DISCERN and PEMAT-P tools are validated instruments, they were originally developed to evaluate traditional patient education materials and may not fully accommodate the unique structural and generative features of AI-produced content. This potential misalignment should be considered when interpreting results.

Future studies should explore user-centered evaluations involving diverse patient populations with varying health literacy levels. Prompt optimization strategies should be tested to enhance actionability and personalization. Additionally, developing domain-specific, guideline-aligned AI models may improve the reliability and relevance of chatbot responses, particularly for complex or rare conditions, such as sarcoidosis. Longitudinal assessments and real-world implementation studies are warranted to evaluate their clinical impact.

## 5. Conclusions

AI chatbots hold promise for enhancing patient education in complex and underrepresented conditions, such as sarcoidosis. This study demonstrates that, while certain models, particularly those with retrieval-augmented architectures, can generate highly accurate and structured information, they often lack accessibility and actionable content. Most responses remain difficult to understand and are insufficiently tailored to patients with varying levels of health literacy.

To bridge this gap, future chatbot development should move beyond accuracy alone and integrate health literacy adaptation, prompt refinement, and clinical applicability. Tailoring outputs to diverse user needs and embedding evidence-based content into conversational design are crucial. Without these enhancements, AI chatbots risk exacerbating existing disparities in digital health communication rather than mitigating them.

## Figures and Tables

**Table 1:** Overview of the evaluation metrics used in the study.

Metric	Purpose	Scoring Range	Interpretation of Scores
DISCERN	Evaluates the reliability and quality of written health information, especially regarding treatment choices.	16–80	≥63: Excellent 51–62: Good 39–50: Fair <39: Poor
PEMAT-P (Patient Education Materials Assessment Tool—Printable)	Assesses the understandability and actionability of patient education materials.	0–100% (two subscores)	≥70%: Acceptable
WRR (Web Resource Rating)	Measures the structural quality and transparency of digital health content.	0–15	≥11: High quality 6–10: Moderate <6: Low
Flesch–Kincaid Grade Level (FKGL)	Estimates the U.S. school grade level required for a reader to comprehend the text.	Grade level	≤8: Suitable for general public
Flesch Reading Ease (FRE)	Assesses overall readability based on sentence and word length.	0–100	≥60: Easy to read 30–59: Difficult <30: Very difficult

**Table 2:** Summary of AI chatbot performance across evaluation scales.

Chatbot	DISCERN Score	PEMAT-P Score	WRR Score
ChatGPT-4o	70.06 ± 3.85	77.75 ± 4.62	70.78 ± 5.47
ChatGPT-4o Deep Search	74.20 ± 2.92	79.33 ± 3.14	71.24 ± 4.11
Perplexity	68.75 ± 3.25	76.38 ± 4.19	82.03 ± 2.90
Grok3 Deep Search	63.56 ± 4.77	82.25 ± 3.26	70.63 ± 3.41
DeepSeek	56.20 [51.00–62.00]	69.50 [64.00–73.00]	62.10 [57.00–66.00]
Gemini Flash	51.30 [45.00–57.00]	66.80 [61.00–72.00]	60.90 [55.00–65.00]
Grok3	58.25 ± 4.01	67.90 ± 3.72	59.75 ± 3.38
Copilot	54.12 ± 3.94	65.88 ± 4.16	58.33 ± 4.25
ChatGPT-3.5	53.00 [47.00–59.00]	63.40 [60.00–67.00]	55.80 [50.00–61.00]
Perplexity Research	66.42 ± 3.27	75.70 ± 4.03	79.55 ± 4.67
DeepSeek Deep Think	61.75 ± 4.12	70.25 ± 3.88	68.90 ± 3.56

WRR, Web Resource Rating; PEMAT-P, Patient Education Materials Assessment Tool for Printable Materials; DISCERN, Quality Criteria for Consumer Health Information. Scores exhibiting normal distribution are presented as mean ± standard deviation, while those not meeting the assumption of normality are reported as median [minimum–maximum].

**Table 3:** Advantages and disadvantages of AI chatbots in generating sarcoidosis-related patient education content, based on DISCERN, PEMAT-P, WRR, and readability assessments from this study.

AI Chatbot	Advantages	Disadvantages
ChatGPT-4	Moderate reliability and readability; concise responses	Limited actionability; responses lack detailed guidance
ChatGPT-4o	Improved content structure over ChatGPT-4; moderate understandability	Low WRR and actionability; readability remains high
ChatGPT-4o Deep Research	Highest reliability and actionability; structured, evidence-based content	Very low readability; requires advanced health literacy
Copilot	Readable and brief; low complexity, suitable for general users	Lowest performance overall; lacks accuracy and depth
DeepSeek	Moderate scores across all domains; balanced output	Limited actionability; moderate readability
DeepSeek Deep Think	Consistent reliability and good understandability; balanced design	Requires high reading level; lacks actionable steps
Gemini2 Flash	High readability; accessible to users with lower literacy	Low reliability and WRR; shallow content
Grok3	Readable; understandable format for basic queries	Limited depth and actionability despite high readability
Grok3 Deep Search	High reliability and actionability; detailed, structured responses	Low readability; not suitable for the general public
Perplexity	Good balance of content depth and structure	High reading level: responses may overwhelm users
Perplexity Research	Very high WRR and DISCERN scores; well structured and informative	Lowest readability; inaccessible to average patient

## Data Availability

Data are contained within the article.
